# Case report: a camouflaged parathyroid carcinoma with initial misdiagnosis

**DOI:** 10.1186/s12893-019-0638-x

**Published:** 2019-11-21

**Authors:** Hongtao Cao, Weibin Wang

**Affiliations:** Department of General Surgery, Peking Union Medical College Hospital, Chinese Academy of Medical Sciences/Peking Union Medical College, Beijing, 100730 China

**Keywords:** Parathyroid adenocarcinoma,, intrathyroidal,, asymptomatic,, normocalcemic

## Abstract

**Background:**

Parathyroid carcinoma is a rare malignancy with an increasing incidence. Most patients are characterized by the presence of severe primary hyperparathyroidism, especially hypercalcemia, while patients with normal level of serum calcium are extremely rare. Unfortunately, patients free of hypercalcemia are usually diagnosed at a later stage and suffer from a rather poor prognosis.

**Case presentation:**

We describe a patient diagnosed with intrathyroidal normocalcemic parathyroid carcinoma, whose preoperative ultrasonography suggests that the tumor is located inside the thyroid gland and present without obvious clinical manifestations, which makes it more challenging for diagnosis.

**Conclusions:**

Preoperative suspicion of malignancy is of great importance for advanced management while preoperative diagnosis is rather challenging with the limited contribution of imaging examinations. Any abnormality in serum level of calcium or parathormone may help to make an initial diagnosis especially when the level is extremely high. We introduce this case of initial misdiagnosis of an intrathyroidal parathyroid carcinoma, mimicking a suspicious thyroid nodule, to focus on the possible anomalous presentations of this rare condition and on its optimal management.

## Background

Parathyroid carcinoma (PC) is an exceedingly rare malignant endocrine neoplasm with an increasing incidence rate from 3.58 to 5.73 per 10 million people according to SEER (Surveillance, Epidemiology, and End Results) database from the year of 1988 to 2003, and accounts for less than 1% cases of primary hyperparathyroidism [1]. Most PCs occur in a single inferior parathyroid gland [2], only very few occur inside the thyroid because merely 0.2% parathyroid glands undergo an abnormal embryological migration from the third and fourth branchia larches [3]. The preoperative diagnosis of intrathyroidal PC is difficult, especially when there are no obvious clinical evidences, such as palpable cervical mass, hyperparathyroidism, hypercalcemia or osteoporosis, which make it much more challenging for diagnosis and further management. Therefore, we report an unusual case of intrathyroidal asymptomatic parathyroid carcinoma with normal level of serum calcium, which to the best of our knowledge, has never been reported in literature ever before.

## Case presentation

**Fig. 1:**
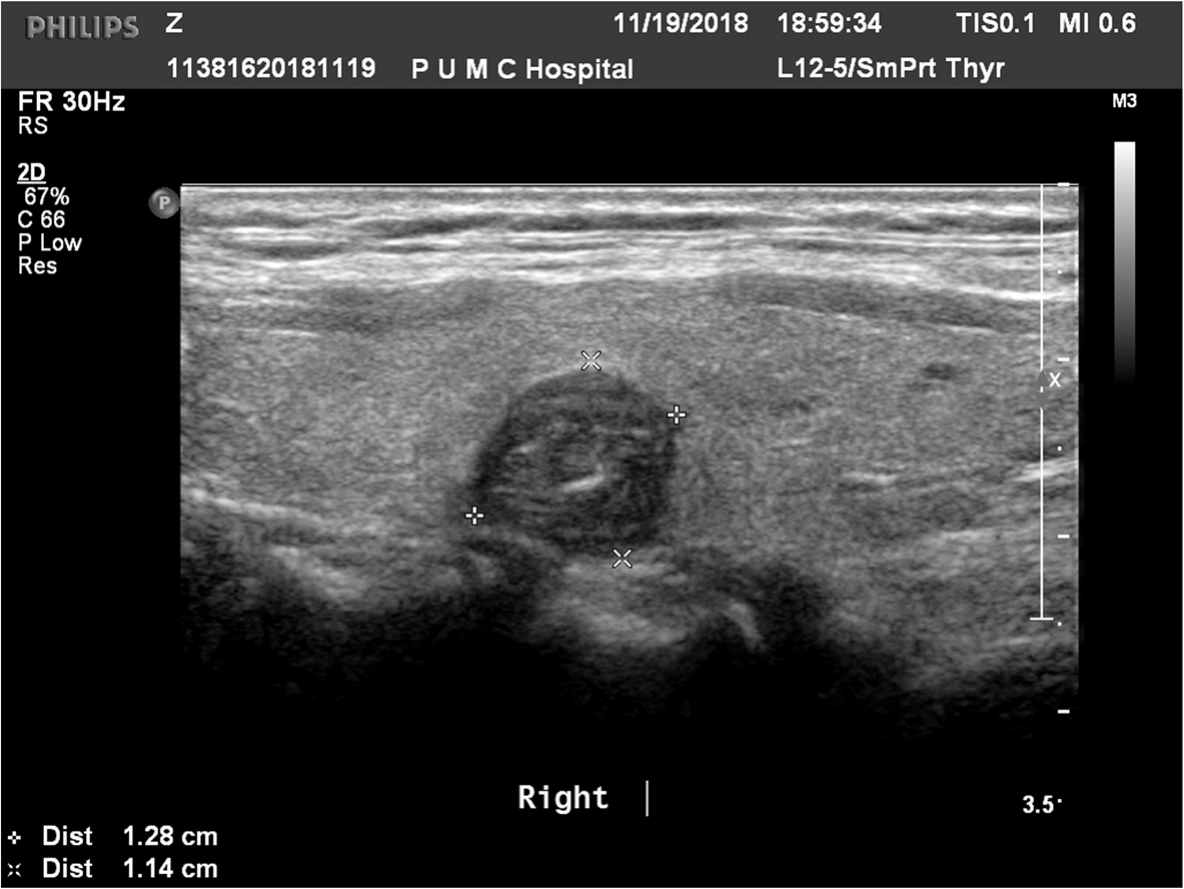
Ultrasonography of the parathyroid carcinoma

A 56-year-old previously healthy Chinese female was admitted to our medical center because of detection of a malignancy-suspected nodule in the right lobe of the thyroid by a routine ultrasonography examination two months ago, which displayed a 1.28 cm*1.14 cm hypoechoic nodule inside the right lobe of thyroid with irregular shape and fairly clear margins close to the posterior capsule, and the Color-flow Doppler imaging showed short-trip blood flows inside (Fig. 1). There were no complaints of palpitation, hyperhidrosis, hoarseness, dyspnea, dysphagia, osteoporosis or convulsion of limbs. The patient was soon scheduled for surgery, before which a preoperative assay of level of serum calcium (Ca) and phosphorus (P) showed normal (Ca 2.50 mmol per liter, P 0.94 mmol per liter).

During the operation, it was found that the tumor was completely located inside the right lobe of thyroid, the texture of which was soft, and the thyroid membrane was intact while no infiltration of adjacent structures was seen. The intraoperative rapid pathology highly suspected malignancy while no characteristic papillary pathological structure was detected, remaining further classification still in need of postoperative paraffin pathology. In addition, during the operation, we detected that some right VI lymph nodes were abnormal swell and hyperplasia, consequently, we finally performed right thyroid lobectomy combined with right VI lymphadenectomy.

**Fig. 2:**
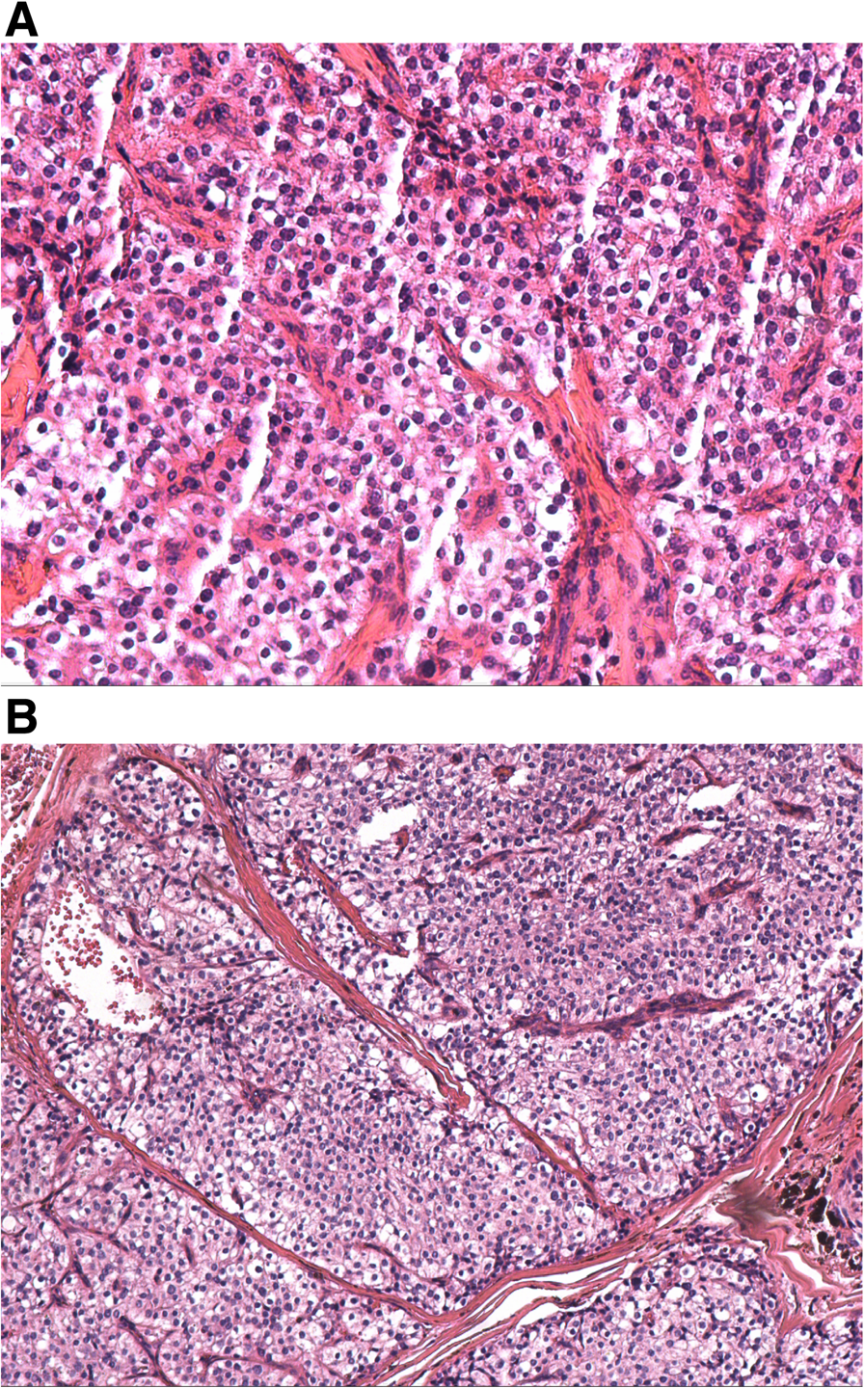
**a** & **b** pathology of the parathyroid carcinoma

The patient was sent back to ward in generally good condition after a smooth surgery and an immediate postoperative assay of calcitonin (CT) and carcinoembryonic(CEA)antigen showed no abnormality (CT < 1.5 pg per milliliter, CEA 2.32 ng per milliliter). After three days of medical care, the patient recovered well and was discharged smoothly. The postoperative paraffin pathological diagnosis revealed adenocarcinoma of parathyroid (Fig. 2, A&B)and the level of serum parathormone (PTH), Ca and P showed no abnormality (PTH 66.1 pg per milliliter, Ca 2.31 mmol per liter, P 1.17 mmol per liter). In addition, 99 m-Tc-MIBI-Pertechnetate imaging showed that the rest parathyroid appeared no abnormality. At a six-month follow up visit after operation, no discomforts or side effects were complained by the patient as well as no obvious locally recurrence were indicated by ultrasonography and the patient is still under regular routine follow up.

## Discussion and conclusions

PC is a rare malignancy accounting for less than 1% cases of primary hyperparathyroidism. The peak age of incidence is from 50 to 60 [4], and both genders are equally affected. The etiology of PC still remains unclear, while mutations of CDC73 gene, encoding parafibromin, are reported to be the most frequent genetic alterations, as well as mutations of MEN1, RET, RB1, TP53, PRUNE2 gene, alterations of PI3K/AKT/mTOR pathway, microRNA profile and methylation pattern have also been identified in PCs [5, 6]. PC, generally a functioning neoplasm, tends to present with manifestations associated with severe hyperparathyroidism, such as osteoporosis, renal involvement, and even hypercalcemia crisis, in addition, uncharacteristic symptoms like fatigue, weight loss, nausea, vomiting, abdominal pain, polyuria, and polydipsia can also occur due to hypercalcemia. Asymptomatic PC is rather rare, only compromising approximately 2% of all [7], and often fails to be detected unless symptoms such as palpable cervical mass, hoarseness or dysphagia [8] appear resulting from local growth or aggressive invasion.

In absence of reliable consensual clinical diagnostic criteria, preoperative diagnosis of PC is still difficult. Consequently, final diagnosis depends on postoperative histological and pathological examination. However, to distinguish PC from benign lesions, especially atypical adenomas is still challenging. Pathological features such as diffuse growth pattern, thickened fibrous band or fibrous septa, mitotic activity and invasion of capsular or vascular highly indicate malignancy. To be more specifically, unequivocal evidence of adjacent tissues infiltration, vascular or perineural space invasion and especially lymph or distal metastases will make it more credibly to be diagnosed with PC [9, 10]. In this case, it was clearly found that neoplastic cells were crowdedly arranged into nests, divided into irregular lobules by thickened fibrous septa and extended over the fibrous capsule, invaded into vascular lumen and vessel walls, which made it more well-defined to indicate malignancy.

Radical surgery, which requires an en-bloc resection of the tumor and an ipsilateral thyroid lobectomy with gross clear margins as well as remove of involved structures or local metastatic lymph nodes, remains the primary and most effective management modality in the treatment of PC, before which severe hypercalcemia should be controlled [11]. Postoperative assay of serum calcium and PTH level is a simple and important evidence for evaluating the therapeutic efficacy of treatment and predicting the recurrence of tumor as well. However, when referring to asymptomatic PC, a routine postoperative imaging detection such as ultrasonography, computed tomography scan, 99 m-Tc-MIBI-Pertechnetate imaging, 18F-fluorodeoxyglucose (FDG) positron emission tomography-computed tomography and even 18F-fluorocholine (FCH) positron emission tomography-computed tomography will function well [12, 13]. For patients with florid metastatic disease who are less likely to derive a large benefit from local surgical resection, management of hyperparathyroidism is fundamental, since parathyroid cancer is usually radioresistant, moreover, insufficient standard chemotherapy regimen is available [11]. Surgery is supposed to be the only effectively curable method for patients with symptomatic primary hyperparathyroidism, especially suspected parathyroid carcinoma. As for secondary hyperparathyroidism, surgical treatment also remains effective in case of medical treatment failure [14], and total parathyroidectomy combined with parathyroid muscular or subcutaneous auto-implantation is likely to provide beneficial effects [15].

## Data Availability

All the data and material are from the patient’s assay and examination of Peking Union Medical College University Hospital, which are real, credible and for availability .

## Abbreviations

Ca: Calcium
CEA: Carcinoembryonic
CT: Calcitonin
FCH: 18F-fluorocholine
FDG: 18F-fluorodeoxyglucose
P: Phosphorus
PC: Parathyroid carcinoma
PTH: Parathormone
SEER: Surveillance, Epidemiology, and End Results
